# Intestinal epithelium integrity after delayed onset of nutrition in broiler chickens

**DOI:** 10.1016/j.psj.2020.08.079

**Published:** 2020-09-12

**Authors:** M.S. Hollemans, J. van Baal, G. de Vries Reilingh, B. Kemp, A. Lammers, S. de Vries

**Affiliations:** ∗ Innovation Team, Coppens Diervoeding B.V., NL-5700AB Helmond, The Netherlands; † Adaptation Physiology Group, Wageningen University & Research, NL-6700AH Wageningen, The Netherlands; ‡ Animal Nutrition Group, Wageningen University & Research, NL-6700AH Wageningen, The Netherlands

**Keywords:** delayed nutrition, intestinal integrity, gene expression, tight junction, broiler

## Abstract

Fasting older broiler chickens (>7 d of age) enlarges the intestinal tight junction (**TJ**) pore size, resulting in high paracellular intestinal permeability. Broiler chickens often do not receive feed and water (nutrition) directly after hatch, which may result in fasting up to 72 h of age. Whether perinatal fasting affects intestinal permeability is minimally studied. We therefore investigated whether delayed access to nutrition after hatch increases intestinal permeability, compared with broilers receiving early access to nutrition. Therefore, 432 hatched broilers received nutrition 72 h after hatch (delayed nutrition [**DN**]) or directly after hatch (early nutrition [**EN**]) and were reared under similar conditions until 14 d of age. Two hours after application of an oral pulse dose (3.85 mg) of fluorescein isothiocyanate-dextran (4000 Da) at 4, 10, and 14 d of age, blood plasma concentrations of the marker were measured in 24 to 36 broilers per treatment and time point. Marker concentration in plasma did not differ between DN and EN broilers at any age. The villus width measured in at least 8 broilers per treatment was smaller in DN than in EN broilers at 4 d for both the ileum (92 ± 3 μm vs. 121 ± 4; *P* < 0.001) and colon (100 ± 3 vs. 120 ± 4; *P* < 0.01). Real-time quantitative PCR revealed that the expression of TJ protein claudin 3 in the ceca was elevated in DN, compared with EN broilers at 4 d of age, whereas that of zonula occludens 1 in the ileum was reduced. Expression of host defense-related genes was reduced in DN, compared with EN broilers, in the ileum (cyclo-oxygenase 2, mucin 2) and ceca (interleukin 1β, cyclo-oxygenase 2). We conclude that 72-hour DN reduced the BW up to 14 d of age, coinciding with transient effects on the villus width in the ileum and colon, and divergent expression of genes involved in TJ formation and host defense. These effects likely reflect the delayed onset of intestinal and immune development in DN, compared with EN broilers, while DN does not fundamentally alter intestinal permeability.

## Introduction

Newly hatched broilers in conventional hatcheries can experience a delay in access to feed and water up to 72 h from hatch (delayed nutrition [**DN**]), caused by a combination of spread in individual hatch moments, hatchery procedures, and transport duration to the farm. Previous studies observed that broilers receiving DN have a lower BW from farm placement onward than broilers receiving early nutrition (**EN**) (reviewed by de Jong et al., 2017). In addition, the intestinal length and weight, villus size, absorptive area, and thickness of the mucus layer in the duodenal, jejunal, and ileal tissue were smaller in DN than in EN broilers (e.g. Smirnov et al., 2004; Lamot et al., 2014). These data suggest that DN, compared with EN, delays intestinal development in broilers, but it is unclear whether severe DN up to 72 h hampers integrity of the intestinal epithelium, allowing for excessive paracellular transport. This may facilitate translocation of intestinal bacteria and microbe-associated molecular patterns, causing (chronic) inflammation resulting in reduced feed efficiency. Furthermore, bacterial translocation is suggested to result in lameness in broilers, as translocated bacteria may adhere to the cartilage of femora and tibiae, causing chondronecrosis (Wideman et al., 2012).

Intestinal epithelial cells are sealed together by tight junctions (**TJ**), which dynamically regulate paracellular transport of molecules from the intestinal lumen through the epithelium into the bloodstream. Tight junctions are composed of transmembrane proteins in which claudins (**CLD**) and junctional adhesion molecules (**JAM**) are suggested to form a physical barrier (Schneeberger and Lynch, 2004; Anderson and Van Itallie, 2009; Turner, 2009). Within this network of proteins, pores are present to facilitate transport of water, ions, and small nutrients. Zonula occludens (**ZO**) are peripheral scaffold proteins, linking the TJ complex to the actin cytoskeleton. In chicken, the TJ are already established and functional at hatch (Karcher and Applegate, 2008; Ozden et al., 2010).

Fasting of broilers at 7 d of age and older was found to increase intestinal permeability (**IP**), likely via a greater TJ pore size (Kuttappan et al., 2015b; Vicuña et al., 2015b; Gilani et al., 2017b). In mammals, it is hypothesized that stressors elevate cortisol levels, subsequently increasing the TJ pore size to raise influx of nutrients that are required to meet increased metabolic demands (De Punder and Pruimboom, 2015). Broilers subjected to 32-hour DN had greater blood corticosterone levels than EN broilers (van de Ven et al., 2013). This may suggest that DN induces a stress response, increasing IP, to ultimately facilitate nutrient uptake. Unfortunately, this strategy may also enhance paracellular translocation of bacteria and microbe-associated molecular patterns via the TJ into the bloodstream (De Punder and Pruimboom, 2015).

Although a recent study (Gilani et al., 2018) did not show excessive IP in broilers subjected to 24-hour DN, the effects of longer feed and water withdrawal periods on IP are unknown, whereas in common broiler husbandry systems broilers may be withheld from feed and water for up to 72 h. The objective of this study was therefore to evaluate differences between severe DN (72 h) and EN on the intestinal morphology, paracellular transport, and expression of several TJ and IP-regulatory genes in the ileum and ceca, to better understand the effects of DN on IP in broilers.

## Materials and methods

### Experimental Design

The experimental design and procedures were ethically approved according to Dutch law under application number AVD104002016441. This article describes part of a larger study testing effects of EN vs. DN (72 h), and 3 dietary treatments starting from 4 d after hatch onward, in a 2 × 3 factorial arrangement. Data from dietary treatments were pooled because no dietary treatment effects were observed (*P* > 0.10). To account for effects of hatch moment, EN and DN groups were divided into either early (first 12 h of the hatch window) or late (second 12 h of the hatch window) hatchers. Ages are expressed as biological ages (Careghi et al., 2005) in days after hatch throughout the article.

### Animals and Nutrition

Seventeen days incubated ROSS 308 eggs (n = 477) were obtained from a commercial hatchery, transferred to the experimental facility, and placed in HatchCare baskets (HatchTech B.V., Veenendaal, The Netherlands). Eggs were hatched in a climate respiration cell with observed room temperature of 35.7 ± 0.1°C and relative humidity of 55.7 ± 0.1%. From the moment of first hatch, hatched broiler chicks were collected every 3 h and weighed, checked for absence of abnormalities, feather-sexed, and neck-tagged for individual identification. First and last 5% of hatchers, and broilers with abnormalities, were excluded from the experiment and culled. Broilers within each 3-hour hatch block were alternately assigned to DN or EN treatments and placed back in the climate respiration cell for 3 d in baskets containing either feed and flowing water (EN) or not (DN). This procedure was repeated every 3 h resulting in a total of 8 hatch groups within a time window of 3 h (total hatch window: 24 h). Hatch groups were pooled into early (0–12 h) or late hatchers (12–24 h). The average observed room temperature during holding was 34.4°C ± 0.4°C, and relative humidity was 55.8 ± 0.2%. Seventy-two hours after hatch, 432 broilers were weighed and placed in 72 floor pens (3 males and 3 females per pen) until 14 d of age (the end of the experiment), and the surplus of broilers was culled. From placement onward, all broilers had ad libitum access to water and a starter diet (Supplementary Table 1, Supplementary material).

### Body Weight

All broilers were individually weighed within 3 h after hatch (0 d) and at 3 d of age. At 4, 10, and 14 d, 2 broilers per pen were weighed. Of these 2 broilers, the first was selected for organ collection, and the second for repeated measurements on IP as described hereafter.

### In Vivo IP

Intestinal paracellular transport of 4000-Da fluorescein isothiocyanate-dextran (**FITC-d**), reflecting the paracellular transport, was measured by blood plasma concentration of FITC-d after an oral pulse dose of FITC-d, according to previous studies (Vicuña et al., 2015b; Gilani et al., 2018). Briefly, at 4, 10 and 14 d of age, 2 broilers per pen received an oral dosage of 3.85-mg FITC-d (4000 Da, Sigma-Aldrich CO, St. Louis, MO) dissolved in 0.35 mL of PBS. After 2 h, one broiler was taken from the pen and weighed, and blood (0.4 mL) was collected from the jugular vein using heparin-flushed needles and syringes. After blood collection, the broiler was placed back in the pen for repeated procedures at 10 and 14 d of age. Blood was centrifuged directly after sampling (12,000 × *g*, 2 min), and 100 μL of plasma was transferred to black 96-well plates and stored in the dark at −20°C pending analyses. After thawing, fluorescence was measured at an excitation wavelength of 485 nm and an emission wavelength of 528 nm on a Spectramax M5 Multi-Mode Microplate Reader (Molecular Devices, Sunnyvale, CA). A linear standard curve was developed by adding known amounts of FITC-d to naive broiler blood plasma. An 8-step dilution series was made ranging from 1,000 to 2 μg/mL with 4 replicates per dilution. A standard linear curve was fitted on optical density as a function of dilution (R^2^ = 0.98). Levels of FITC-d in plasma were expressed in μg/mL plasma. The detection limit was set at 2.4 μg/mL (mean + 2 × SD) based on naive plasma.

### Organ Collection

Selected broilers were weighed and subsequently euthanized by decapitation for organ collection. An ileal midsection of approximately 2 cm was collected between the Meckel's diverticulum and the ileocecal junction, as well as the whole colon, and one randomly selected cecum from in total 12 broilers per treatment per age (4, 10, 14 d). Intestinal sections were laterally opened, and after removing intestinal contents, Swiss rolls (Moolenbeek and Ruitenberg, 1981) were made and directly frozen in liquid nitrogen and stored at −80°C. From broilers dissected at 4 d, residual yolk was collected and weighed.

### Villus Width

A subset of collected ileum and colon tissues was selected with the random sampling procedure in base R, version 3.5.0. (R Development Core Team, 2018), from both DN and EN broilers. Swiss rolls from the ileum and colon were cut with a microtome at −20°C (7 μm), and 6 sequential tissue sections were mounted on a glass slide and stained with hematoxylin and eosin. The software package LAS X (Leica Microsystems B.V., Amsterdam, The Netherlands) was used to measure the villus width. The villus height could not be measured because of shrinkage of villi after freezing. The villus width was measured from at least 5 villi from 6 sections, resulting in 30 measurements per slide that were averaged per sample.

### RNA Isolation and cDNA Synthesis

A total of 10 ileal and 10 cecal tissue samples per treatment group (n = 40), obtained at 4 d of age, was randomly selected by the random sampling procedure in base R. Approximately 100 mg of tissue was ground in liquid nitrogen, and the total RNA was extracted using TRIzol reagent (Thermo Fisher Scientific, Bleiswijk, The Netherlands) following the manufacturer's instructions. After extraction, genomic DNA was eliminated by an on-column DNase digestion step using an RNeasy kit (Qiagen, Venlo, The Netherlands). The concentration and purity of the RNA was determined using a NanoDrop ND-1000 (Thermo Fisher Scientific). Integrity of the RNA was evaluated using a Bioanalyzer 2100 and RNA 6000 Nano LabChip kit (Agilent, Santa Clara, CA) revealing RNA integrity values ranging from 9 to 10. Four cecal tissues displaying a too low RNA concentration were discarded, resulting in a total of 36 samples for further analyses. For first-strand cDNA synthesis, 200-ng RNA was reverse-transcribed with Superscript III Reverse Transcriptase (200 U; Thermo Fisher Scientific) in the presence of random hexamer primers (250 ng; Roche Diagnostics, The Netherlands), DL-dithiothreitol (10 mM), and dNTP (1 mmol), in a 20-μL reaction volume at 50°C for 60 min, followed by 55°C for 15 min. Reactions were terminated at 70°C (15 min). On real-time quantitative PCR analysis, cDNA was stored at −20°C.

### Real-Time Quantitative PCR

Real-time quantitative PCR was performed with a QuantStudio 5 qPCR system (Thermo Fisher Scientific) using the SensiFAST SYBR Lo-ROX kit (Bioline, London, UK), following the manufacturer's instructions. Primers were designed with Primer Express Software (Life Technologies, Bleiswijk, The Netherlands), and recommended primer sets that span an intron were selected (Table 1). Amplification conditions consisted of 95°C for 2 min, followed by 40 cycles (95°C for 15 s followed by 60°C for 30 s). A final melting protocol with ramping from 60°C to 95°C with 0.1°C increments/s confirmed PCR specificity. Absolute quantitative mRNA measurement was performed by establishing a linear calibration curve using 10-fold serial dilutions of cDNA template for corresponding genes. Abundance of RNA was normalized to that of ribosomal protein lateral stalk subunit P0 because the NormFinder algorithm (Andersen et al., 2004) pointed out this gene was most stably expressed among our samples compared with β-actin and glyceraldehyde-3-phosphate dehydrogenase.

**Table 1:** Chicken gene-specific primers used for RT-qPCR.

Gene 1	Accession number	Efficiency (%)	Amplicon size (bp)	Primers	
				Forward	Reverse
COX-2	NM_001167719	87	127	5′-ATTCCTGACCCACAAGGCAC-3′	5′-AGTCAACCCCATGGCCGTAA-3′
ZO-1 2	XM_015278975	90	63	5′-CCGCAGTCGTTCACGATCT-3′	5′-GGAGAATGTCTGGAATGGTCTGA-3′
JAM-2 2	NM_001006257	93	59	5′-AGCCTCAAATGGGATTGGATT-3′	5′-CATCAACTTGCATTCGCTTCA-3′
MUC-2	NM_001318434	90	214	5′-ATTGAAGCCAGCAATGGTGT-3′	5′-TGACATCAGGGCACACAGAT-3′
CLD-3	NM_204202	87	159	5′-TATGGGGCTGGAGATCGGT-3′	5′-ACCACGCAGTTCATCCACAG-3′
IL-1β	HQ329098	98	215	5′-GACATCTTCGACATCAACCAG-3′	5′-CCGCTCATCACACACGACAT-3′
ACTB	NM_205518	96	162	5′-GCCCTGGCACCTAGCACAAT-3′	5′-GCGGTGGACAATGGAGGGT-3′
GAPDH	NM_204305	100	135	5′-ATCCCTGAGCTGAATGGGAAG-3′	5′-AGCAGCCTTCACTACCCTCT-3′
RPL-P0 2	NM_204987	93	83	5′-TTGGGCATCACCACAAAGATT-3′	5′-CCCACTTTGTCTCCGGTCTTAA-3′

1 COX-2: cyclo-oxygenase 2; ZO-1: zonula occludens 1; JAM-2: junctional adhesion molecule 2; MUC-2: mucin 2; CLD-3: claudin 3; IL-1β: interleukin 1β; ACTB: actin β; GAPDH: glyceraldehyde-3-phosphate dehydrogenase; RPL-P0: ribosomal protein lateral stalk subunit P0.

2 The following primers were taken from literature: ZO-1 and JAM-2 (Chen et al., 2015) and RPL-P0 (Staines et al., 2016).

### Statistical Analyses

All data were processed, statistically analyzed, and presented using R, version 3.5.0. General linear models were established using the nlme package (Pinheiro et al., 2018) to estimate effects of DN and EN per time point on the BW (day 3, 4, 10, and 14), villus width (day 4, 10, and 14), and absolute and relative yolk weight (day 4), with treatment (DN, EN), hatch moment (early, late), and their two-way interaction as fixed effects. Gene expression at day 4 was estimated for each gene within the organ (the ileum and ceca), with a general linear model using treatment as a fixed effect. Model fits were assessed by normality and homoscedasticity of residuals by qq plots, plotting residuals against fitted values, and if needed by testing likelihood ratios between models (Pinheiro and Bates, 2000). Logarithmic transformation was performed on the following dependent variables: BW at day 3, villus width, and residual yolk weights. Data from one broiler that represented a biological impossible value (DN; day 4) for the BW and residual yolk were excluded from further analyses. Data from FITC-d plasma levels (day 4, 10, and 14), and gene expression of zonula occludens 1 (ZO-1) in the ileum, did not meet model assumptions of homoscedasticity and were therefore analyzed with a Kruskal-Wallis test. All data are represented as (back-transformed) estimated marginal means ± SEM unless stated otherwise. Differences among means were considered significant if *P* ≤ 0.05, and statistical tendencies were considered if *P* ≤ 0.10.

## Results

### Broiler Performance Parameters

At 3 d after hatch, DN broilers had a smaller BW than EN broilers (Δ = 30.1 g; *P* < 0.001; Table 2), particularly in early hatchers (feeding x hatch moment *P* < 0.001). Residual feed intake of EN broilers varied between 7.2 and 18.8 g during holding (0–72 h after hatch). The BW was smaller in DN than in EN (*P* < 0.001; Table 3) at day 4 (Δ = 30.0 g), 10 (Δ = 85.0 g), and 14 (Δ = 122 g), without significant effects of hatch moment (data not shown). The absolute weight of the residual yolk was not affected by the hatch moment or by feeding (DN: 0.7 ± 0.09 g; EN: 0.7 ± 0.08 g P = 0.30).

**Table 2:** Effect of early or 72-h delayed nutrition on the BW (g) of broiler chickens at 3 d after hatch, for early- and late-hatched broilers.

Treatments	Mean	SEM	n
Early hatchers 1			
Delayed nutrition	39.4^a^	0.46	115
Early nutrition	71.2^c^	0.47	111
Late hatchers 1			
Delayed nutrition	40.2^a^	0.52	90
Early nutrition	68.5^b^	0.54	85
*P*-values 2			
Feeding × hatch moment	<0.001		
Feeding	<0.001		
Hatch moment	0.07		

^a-c^Data are presented as estimated marginal means with their SEM and the number of broilers within each group (n). The means lacking a common superscript differ (*P* ≤ 0.05).

1 Early hatchers hatched in the first 12 h and late hatchers in the second 12 h of the hatch window. First and last 5% of hatchlings were removed from the study.

2 Model established *P*-values for fixed effect of feeding, hatch moment, and their interaction.

**Table 3:** Effects of early or 72-hour delayed nutrition on the villus width in the ileum and colon and the BW (g), at 4, 10, and 14 d after hatch in broiler chickens.

Treatment	Age (day)	Villus width 1						BW		
		Ileum			Colon					
		Mean	SEM	n	Mean	SEM	n	Mean	SEM	n
	4									
Delayed nutrition		92	3.18	8	100	3.45	9	39.2	0.86	34
Early nutrition		121	3.74	10	120	3.92	10	69.2	0.93	29
Fixed effect 2		<0.001			<0.01			<0.001		
	10									
Delayed nutrition		134	7.33	4	126	6.05	4	175.0	4.19	23
Early nutrition		136	7.44	4	127	6.09	4	260.0	4.19	23
Fixed effect 2		0.92			0.92			<0.001		
	14									
Delayed nutrition		132	8.32	4	132	6.43	4	315.0	8.03	23
Early nutrition		140	8.86	4	149	7.28	4	437.0	7.86	24
Fixed effect 2		0.12			0.12			<0.001		

Data are presented as estimated marginal means with their SEM and the number of broilers within each group (n).

1 Mean width of measurements of 30 villi per broiler in μm.

2 Model established *P*-values for fixed effect of feeding.

### Villus Width and Plasma FITC-d Levels

**Figure 1:**
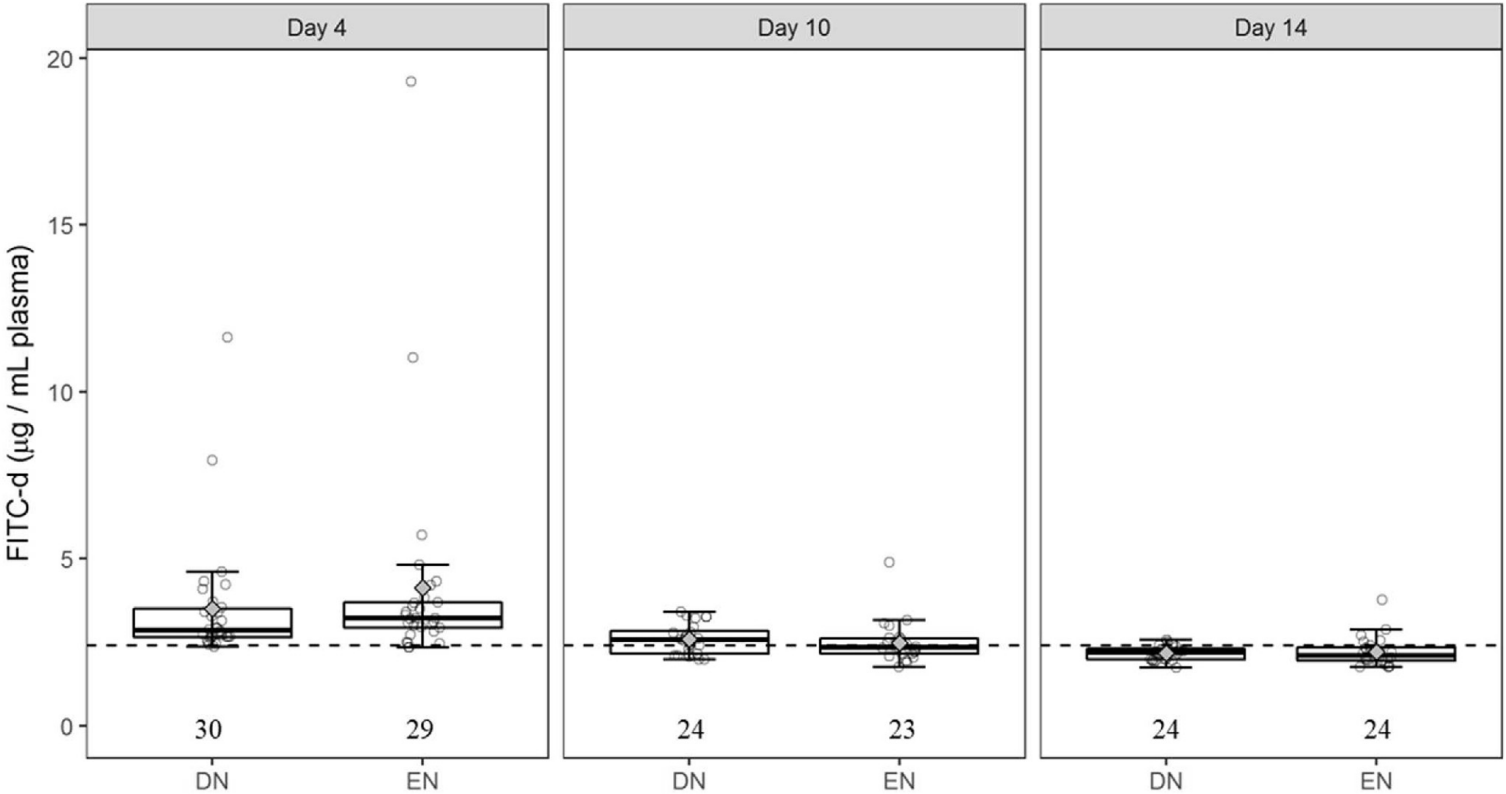
Effects of 72-hour delayed nutrition (DN) or early nutrition (EN) on fluorescein isothiocyanate-dextran (FITC-d) blood plasma concentrations (μg FITC-d/mL) after an oral pulse dose of 3.85 mg FITC-d at 4, 10, and 14 d after hatch in broiler chickens. Circles represent concentrations measured in individual plasma samples. The horizontal line in the boxplots represents the median, and whiskers span the 1.5 ∗ interquartile range from the box. ♦ represents raw group means. The dotted horizontal line indicates the lower detection limit (2.4 μg/mL). No differences among means (*P* > 0.10) were observed. The numbers represent the number of observations for each treatment group for each age.

In DN vs. EN broilers, the villus width was smaller in the ileum (Δ = 29 μm; *P* < 0.001) and colon (Δ = 20 μm; *P* < 0.01), however, no differences were observed at 10 and 14 d (Table 3). Plasma levels of FITC-d at 4 d were not affected by feeding at 4, 10, and 14 d of age (Figure 1). Multiple plasma samples had FITC-d concentrations less than the detection limit at 10 (23 of 47) and 14 d of age (37 of 48), but not at 4 d.

### Gene Expression

**Figure 2:**
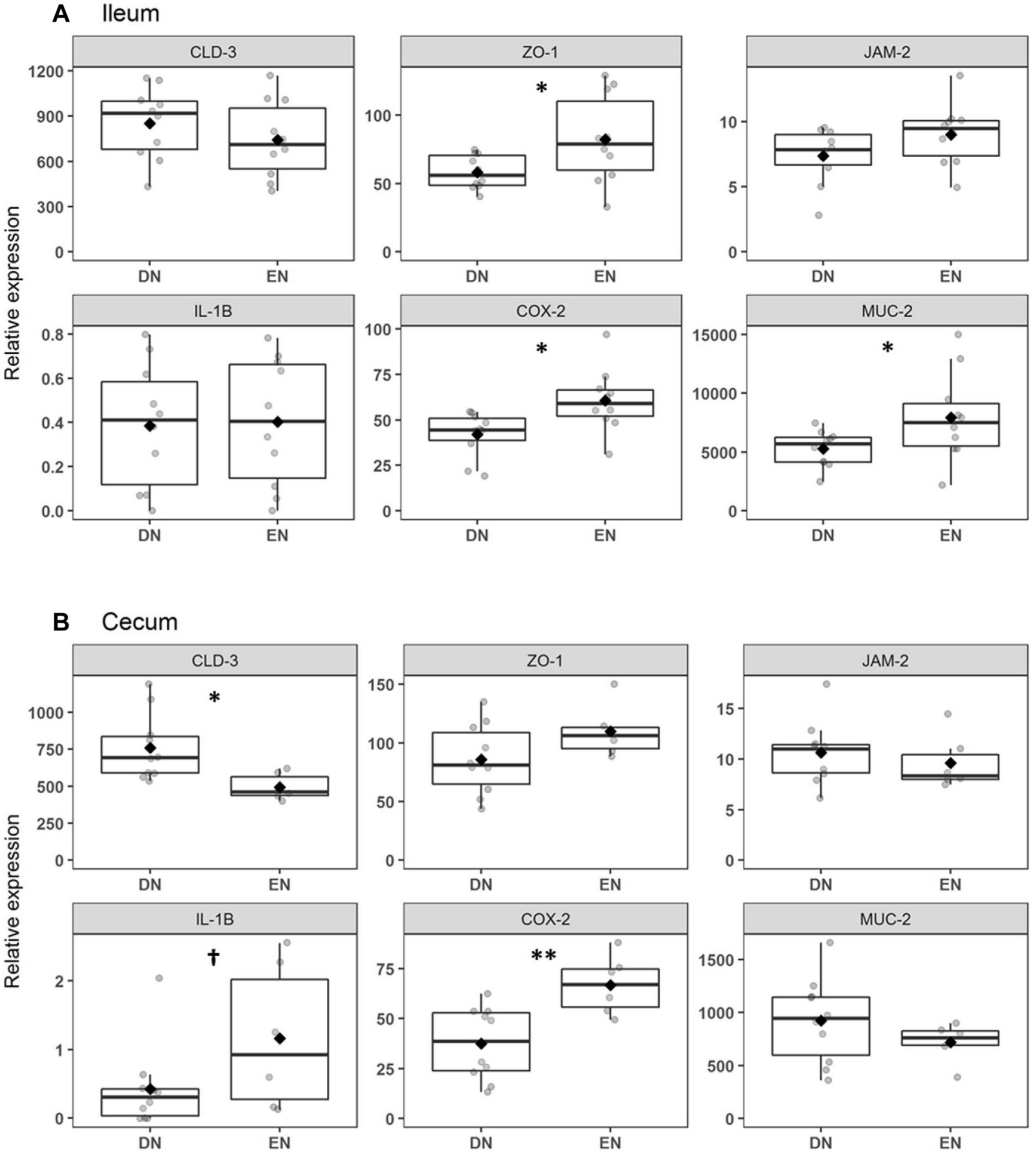
Relative expression levels of genes involved in tight junction formation and organization affected by early nutrition (EN) or 72-h delayed nutrition (DN) in the ileum (A) or cecum (B) at 4 d after hatch in broiler chickens. Absolute mRNA levels were normalized to the corresponding mRNA levels of RPL-P0. Circles represent individual broilers. The horizontal line in the boxplots represent the median, and whiskers span the 1.5 ∗ interquartile range from the box. ♦ represents raw group means. Differences among means are indicated with ∗∗ (*P* ≤ 0.01) or ∗ (*P* ≤ 0.05) and tendencies (*P* ≤ 0.10) with †. For all treatment groups and organs, n = 10, except for the cecum from EN broilers (n = 6). Abbreviations: CLD-3, claudin 3; COX-2, cyclo-oxygenase 2; IL-1β, interleukin 1β; JAM-2, junction adhesion molecule 2; MUC-2, mucin 2; ZO-1, zonula occludens 1.

Real-time quantitative PCR analysis in the ileum and ceca collected at 4 d revealed that gene expression of TJ protein claudin 3 (CLD-3) was greater (*P* = 0.001) in the ceca of DN broilers than that of EN broilers, but not in the ileum (Figure 2). Expression of scaffold protein ZO-1 was lower (*P* = 0.05) in the ileum of DN broilers than in that of EN broilers. The expression of junction adhesion molecule 2 was not affected by treatment in both segments. With respect to genes related to host defense pathways, we found lower gene expression of cyclo-oxygenase 2 (**COX-2**) in both the ileum (*P* = 0.01) and ceca (*P* = 0.003) in DN than in EN broilers. Expression of the cytokine interleukin 1β tended to be lower (*P* = 0.10) in the ceca of DN broilers, whereas the expression of mucin 2 (**MUC-2**) was greater (*P* = 0.05) in the ileum of DN broilers.

## Discussion

The objective of this study was to study effects of 72-hour DN, compared with EN, on intestinal epithelium integrity in broilers, as measured by the villus width and IP. Abundance of the RNA was measured by real-time quantitative PCR to study gene expression of TJ-related genes and expression of genes related to host defense pathways. Our data are in line with the current literature on effects of DN, compared with EN, with regard to the BW, villus width, and IP. We observed a smaller villus width and divergent expression levels of genes involved in TJ formation and organization in the ileum and ceca of DN and EN broilers at 4 d of age but no effects of feeding strategy on IP.

### Effects of DN on Broiler Growth Performance and Intestinal Integrity

The BW was greater in EN than in DN chickens throughout the experiment (77% at 4 d to 39% at 14 d), as expected (de Jong et al., 2017; Hollemans et al., 2018). The residual yolk weight was evaluated to compare yolk disappearance in EN with that in DN broilers. We observed no differences in the residual yolk weight between DN and EN broilers or among hatch moments, suggesting that yolk resources may be sufficient to meet nutrients requirements for periods of DN up to 72 h. The width of intestinal villi was measured to confirm that in our study; DN resulted in impaired intestinal morphology. The villus width was smaller in the ileum (32%) and colon (20%) at 4 d of age in DN than in EN broilers and diminished rapidly on feeding, as expected (Geyra et al., 2001; Lamot et al., 2014). These findings indicate a smaller absorptive area in DN than in EN broilers (Kisielinski et al., 2002).

We are the first to report effects of prolonged DN for 72 h on IP and found that levels of FITC-d in plasma were unaffected by feed and water deprivation after hatch. The absence of treatment effects on FITC-d plasma levels suggests that DN has no, or at least short-lasting, effects on paracellular transport across the intestinal epithelium. This is in accordance with previous findings in broilers subjected to a 24-hour duration of DN (Gilani et al., 2018).

Claudin, JAM, and ZO proteins play an essential role in maintaining intestinal integrity and paracellular transport (Schneeberger and Lynch, 2004; Anderson and Van Itallie, 2009). In a dexamethasone model in broilers that invokes intestinal inflammation, a greater paracellular transport of FITC-d via the intestinal epithelium was found to be associated with a greater gene expression of CLD-3, ZO-1, ZO-2, and occludin, a TJ-associated transmembrane protein, in the ileum (Barekatain et al., 2019). These observations demonstrate a relation between paracellular transport and expression of certain TJ genes. This relation is further demonstrated in murine in vitro models, where greater CLD, JAM, and ZO gene expression resulted in elevated abundance of these proteins (Zhang et al., 2014). For example, greater gene expression of CLD-1, 2, 3, and 4 resulted in greater transepithelial resistance in intestinal cell monolayers (Lu et al., 2013).

To gain insight into the effects of DN on epithelium integrity on the molecular level, we measured expression of genes involved in the construction of TJ (CLD-3, junction adhesion molecule 2, ZO-1). Whereas FITC-d plasma levels were unaffected by our feeding treatments (DN vs. EN), we observed downregulation of CLD-3 and upregulation of ZO-1 in EN broilers. This may either indicate that the FITC-d method did not accurately reflect IP or that IP was unaffected and that our observations on gene expression reflected maturation of the intestinal tract, rather than IP. With regard to maturation, it was found in rodents that the expression of genes from the CLD family changes during the perinatal period (Holmes et al., 2006).

Other studies suggest that metabolites of intestinal bacteria play a role in the maintenance of intestinal integrity by affecting the expression of TJ-related genes (reviewed by Ulluwishewa et al., 2011). The expression of genes related to host defense was also studied, including immune responses (IL-1β, COX-2), and mucin dynamics (MUC-2), which can indirectly control IP (Turner, 2009; Lee, 2015; Volynets et al., 2016). Gene expression levels of COX-2 and IL-1β were lower (*P* ≤ 0.05) in DN than in EN broilers, primarily in the ceca. Remarkably, we observed no differences in IP, whereas elevated levels of COX-2 and IL-1β are known to induce inflammatory processes and are associated with greater IP (Schneeberger and Lynch, 2004; Fredenburgh et al., 2011; Lee, 2015). For example, IL-1β levels were elevated during intestinal inflammation and decreased TJ integrity in CaCo-2 cells (Al-Sadi et al., 2008), probably via activation of myosin light-chain kinase (Turner, 2009). The enzyme COX-2 is responsible for formation of prostanoids and is involved in inflammatory processes (Fredenburgh et al., 2011). Mice deficient in COX-2 were shown to have a greater IP, as COX-2 was found to upregulate expression of occludin, ZO-1, and CLD-1 (Fredenburgh et al., 2011). Mucin 2 is a gel-forming glycoprotein that covers the luminal surface of the gut and was elevated in EN compared with DN broilers. This finding is in accordance with other studies (Uni et al., 2003; Smirnov et al., 2004) and may suggest differences in colonization of the intestinal tract by bacteria (Deplancke and Gaskins, 2001).

In DN broilers, delayed bacterial colonization compared with EN broilers is suggested (Binek et al., 2000), including differences in microbiota composition in the ileum up to 9 d of age (Simon, 2016). In addition, it was suggested that development of gut-associated lymphoid tissue (**GALT**) after first feeding after hatch may be accelerated, likely as a result of antigenic stimulation by intestinal microbiota and feed (Juul-Madsen et al., 2004; Bar-Shira et al., 2005; Bar-Shira and Friedman, 2006; Simon et al., 2014). Bar-Shira and Friedman (2006) suggested that immune stimulation by microbial colonization in the intestinal tract and subsequent upregulated expression of proinflammatory cytokine genes in the duodenum, colon, and ceca during the first days after hatch is responsible for this accelerated development of GALT. We speculate that the observed effects of DN on the ileum and ceca gene expression may reflect delayed development of GALT because of the lack of feed intake and reduced bacterial colonization in DN compared with EN broilers, rather than differences in IP.

### FITC-d Procedure to Measure Paracellular IP

Various methods to quantify IP are described in literature (reviewed by Gilani et al., 2016a; Wells et al., 2017). In this study we have used the FITC-d method to measure paracellular transport, as this a well-known method to evaluate IP in broilers aged between 4 and 38 d of (Kuttappan et al., 2015a, Kuttappan et al., 2015b; Vicuña et al., 2015b; Gilani et al., 2016b, 2017b, 2018; Baxter et al., 2017; Barekatain et al., 2019). However, retrospectively, our results suggest that the method requires further optimization to accurately measure effects of dietary treatments on paracellular transport of FITC-d in young broilers. First, we reported a relatively large number of plasma samples below the detection limit at 10 (23 of 47) and 14 (37 of 48) d of age. We could not compare these values to literature, as detection limits were not reported (Kuttappan et al., 2015a, Kuttappan et al., 2015b; Vicuña et al., 2015b; Gilani et al., 2016b, 2017b, 2018; Baxter et al., 2017; Barekatain et al., 2019). However, our data indicate poor sensitivity of the method for broilers ≥10 d of age, using the dosing protocol according to the aforementioned studies.

In addition, we observed large contrasts in the BW at all ages, as a result of the treatments (DN vs. EN), pointing out the need for BW correction of pulse doses. Remarkably, adjustment of the oral pulse dose of FITC-d for the BW is commonly not reported (Tellez et al., 2014; Kuttappan et al., 2015a; Vicuña et al., 2015b; Gilani et al., 2017a, Gilani et al., 2017b; 2018; Barekatain et al., 2019). When not corrected for the BW, FITC-d levels in blood may be confounded with absorptive capacity of the gastrointestinal tract (**GIT**). Delayed-nutrition broilers are reported to have a smaller GIT than EN broilers (Lamot et al., 2014) and have a lower absorptive area (e.g. Smirnov et al., 2005). This suggests lower absolute levels of paracellular transport of FITC-d in DN than in EN broilers. Apart from differences in the absorptive area, the BW is positively correlated with the blood volume (Medway and Kare, 1959; Kotula and Helbacka, 1966). Owing to their lower BW, DN broilers had a lower blood volume than EN broilers, resulting in a greater dilution of FITC-d plasma levels. Therefore, FITC-recovery in our study was calculated as follows. The FITC-d levels in the plasma were multiplied by the estimated blood volume (Kotula and Helbacka, 1966) and subsequently divided by the amount of FITC-d supplied as oral pulse dose. This resulted in estimated FITC-d recoveries ranging between 0.35 and 2.51% w/v, which is in accordance with existing literature on broilers up to 10 d of age (0.3 to 1.3% w/v) (Tellez et al., 2014, 2015; Vicuña et al., 2015a; Gilani et al., 2018). Baxter et al. (2017) concluded that the accuracy of the FITC-d method can be optimized in 10-day-old broilers by a greater oral pulse dose (up to 8.32 mg FITC-d/kg BW) and earlier collection of blood after the oral pulse dose (1 h after dosing). However, we were not able to determine whether FITC-d recovery after the oral pulse dose was indeed greater in this study than in our study, as the BW was not published.

Second, we also presume that measuring IP using FITC-d in the fed state might be confounded by a lack of standardization of feed intake and gut fill. However, this standardization is also not reported in other studies measuring IP in vivo (Tellez et al., 2014, 2015; Vicuña et al., 2015a; Baxter et al., 2017; Gilani et al., 2018). Variation in the amounts of digesta in the GIT will result variable dilutions of FITC-d in the intestinal lumen, thus affecting the amount of FITC-d that will have contact with the intestinal epithelium. Finally, the 24-hour habituation period after DN in our study, following procedures of Gilani et al. (2018), may have resulted in rapid regeneration of the TJ pore size. As the TJ pore size is known to be highly dynamic (Shen et al., 2008; De Punder and Pruimboom, 2015), this habituation period may have diminished potential effects on FITC-d permeability. In summary, we propose that future studies comparing IP between DN and EN broilers using the FITC-d procedure should (1) adjust the amount of marker supplied as the oral pulse dose for the BW, (2) apply a short period of fasting to standardize gut fill, and (3) omit habituation periods. Nevertheless, our data obtained on 4 d of age, where all samples had plasma FITC-d concentrations above the detection limit, even if corrected for the blood volume in DN vs. EN broilers, did not point out any differences between DN and EN treatments. This confirms that DN has no, or at least short-lasting, effects on paracellular transport across the intestinal epithelium.

## Conclusions

Our data confirm that impaired growth during the first 14 d observed in DN broilers coincides with transient effects on the ileal and colonic villus widths. Paracellular transport through the intestinal epithelium, measured with FITC-d, was unaffected by DN. The divergent expression of the TJ and host defense genes, together with findings on the villus width, may reflect delayed intestinal and immune development in DN compared with EN broilers, while DN does not fundamentally alter IP.
