# Supplementary material for: Intestinal epithelium integrity after delayed onset of nutrition in broiler chickens
Source: Poult Sci. 2020 Sep 12;99(12):6818–27. doi: 10.1016/j.psj.2020.08.079 (PMC7704972; doi:10.1016/j.psj.2020.08.079)
Supplement: Supplementary Table 1 [file mmc1.docx]

# Supplementary material

**Supplementary Table 1**: Ingredient and calculated nutrient composition of control diet provided during the experiment (in % (w/w), as-fed basis, unless indicated otherwise).

|  |  |  |
| --- | --- | --- |
| Ingredients (in %) |  |  |
| Wheat |  | 36.4 |
| Soybean meal |  | 23.2 |
| Extruded full-fat soybeans |  | 10.0 |
| Rye |  | 20.0 |
| Soybean oil |  | 5.9 |
| L-Lysine HCl |  | 0.3 |
| DL-Methionine |  | 0.3 |
| L-Threonine |  | 0.2 |
| Limestone |  | 1.4 |
| Monocalcium phosphate |  | 1.4 |
| Sodium bicarbonate |  | 0.4 |
| Sodium chloride |  | 0.1 |
| Premix^1^ |  | 0.5 |
| NSP enzyme^2^ |  | 0.03 |
|  |  |  |
| Calculated nutrient composition^3^ |  |  |
| Moisture |  | 118 |
| Crude protein^4^ |  | 229 |
| Digestible lysine^5^ |  | 12.0 |
| Digestible methionine + cysteine^5^ |  | 8.6 |
| Digestible threonine^5^ |  | 7.4 |
| Crude fat |  | 84 |
| Crude fiber |  | 25 |
| Ash |  | 65 |
| Starch^6^ |  | 304 |
| DE (kcal/kg) |  | 2850 |
| Calcium |  | 9.5 |
| Phosphorus |  | 6.5 |

^1^ Supplied per kg of diet (as-fed basis): Vitamin A (12,500 IU); D3 (3000 IU); E (16.8 IU); K3 (3.0 mg); B1 (3.0 mg); B2 (7.5 mg); B6 (4.0 mg) ; B12 (7.5 mg); niacin (45.0 mg); panthothenic acid (10.0 mg); biotin (500.0 mg); choline chloride (500.0 mg); Mn (85.0 mg); Zn (90.0 mg); Cu (15.0 mg); Fe (80.0 mg); I (2.0 mg); Se (0.25 mg).

^2^ Commercial bacterial endo-1,3-β-xylanase (Belfeed, Agrimex N.V., Lille, Belgium).

^3^ Calculated based on feed table of CVB (2007) and specified in g / kg unless stated otherwise.

^4^ Conversion factor: 6.25.

^5^ Apparent total tract digestibility.

^6^ Calculated starch as measured with the amyloglucosidase method (ISO 15914).
